# Long Non-coding RNA LINC-PINT Suppresses Cell Proliferation and Migration of Melanoma via Recruiting EZH2

**DOI:** 10.3389/fcell.2019.00350

**Published:** 2019-12-20

**Authors:** Yangfan Xu, Huixue Wang, Fang Li, Ludwig M. Heindl, Xiaoyu He, Jie Yu, Jie Yang, Shengfang Ge, Jing Ruan, Renbing Jia, Xianqun Fan

**Affiliations:** ^1^Department of Ophthalmology, Ninth People’s Hospital, Shanghai Jiao Tong University School of Medicine, Shanghai, China; ^2^Shanghai Key Laboratory of Orbital Diseases and Ocular Oncology, Shanghai, China; ^3^Zentrum für Augenheilkunde, Universität zu Köln, Köln, Germany

**Keywords:** LINC-PINT, melanoma, EZH2, CDK1, CCNA2, AURKA, PCNA

## Abstract

Long non-coding RNAs (lncRNAs) have been identified as crucial regulators in many human cancers. Many lncRNAs show aberrant expression in cancer, and some of them play critical roles in tumor proliferation, invasion, and metastasis. However, the regulatory functions of lncRNAs in melanoma progression remain to be elucidated. We utilized the Real-time PCR methodology to determine the expression of LINC-PINT in melanoma cell lines. To evaluate the effect of LINC-PINT on tumorigenesis of melanoma, we used Cell Counting Kit-8 (CCK8) and colony formation assay. Flow cytometry assay was used to detect the function of LINC-PINT on cell cycle status. PINT-interacting proteins were identified by chromatin isolation using RNA purification (ChIRP). Microarray assay and bioinformatics analysis were used to find the potential target genes of LINC-PINT and the status of LINC-PINT target gene candidate was verified using chromatin immunoprecipitation assay (ChIP). LINC-PINT plays a role in suppressing the tumorigenicity of melanoma, which was further determined by xenograft model assay. LINC-PINT was significantly downregulated in melanoma tissues and cell lines. The overexpression of LINC-PINT in tumor cells resulted in significant tumor growth reduction and migration inhibition in A375, Mum2B and CRMM1 cells. Results based on the *in vivo* xenograft model were further consistent with the *in vitro* findings that LINC-PINT impeded growth and metastasis of melanoma cells. Microarray assay and bioinformatics analysis indicated that CDK1, CCNA2, AURKA, and PCNA were potential targets of LINC-PINT. In conclusion, LINC-PINT inhibits the tumorigenicity of melanoma through recruiting EZH2 to the promoter of its target genes, leading to H3K27 trimethylation and epigenetic silencing of target genes. LINC-PINT may serve as a novel diagnostic and therapeutic target for melanoma.

## Introduction

Melanoma is a malignant tumor that initiates from pigment-producing cells called melanocytes and progresses in a step-wise fashion (Bastian, 2014). It occurs in tissue that contains these cells, including the base of the epidermis, the eye, and the epithelia of the respiratory and urogenital tract (Arozarena and Wellbrock, 2019). Melanoma is exceedingly aggressive, which is based on the high metastatic potential of melanoma cells. Despite recent progresses in melanoma targeted therapies, this malignancy still could not be efficiently managed (Zingg et al., 2015). Although novel therapeutic strategies have been developed over the past few decades (Hersey and Gallagher, 2012; Niezgoda et al., 2015), metastatic melanoma is associated with a poor prognosis (Tsao et al., 2012). Thus, better therapies for melanoma are in urgent need to be established.

Long non-coding RNAs (lncRNAs) are functionally defined as transcripts more than 200 nucleotides in length with no protein coding potential. There’re tens of thousands lncRNAs in human cells, many of which are uniquely expressed in differentiated tissues or specific cancer types (Schmitt and Chang, 2016; Arun et al., 2018; Wang et al., 2019). LncRNAs are being increasingly recognized to contribute to many biological processes through diverse mechanisms (Cech and Steitz, 2014; Takahashi et al., 2014). Recently, it is reported that many lncRNAs are affecting gene activity in potent *cis-* and *trans-*regulation pattern and they function as scaffolds for chromatin-modifying complexes (Kim and Sung, 2012), thus regulating the process of RNA degradation and histone modifications (Guttman and Rinn, 2012). LINC-PINT, which is also known as long intergenic non-protein-coding RNA p53-induced transcript, has been reported to exert its functions in some diseases. For example, it directly interacts with the polycomb repressive complex 2 (PRC2) to regulate the expression of its target genes (Marin-Bejar et al., 2013, 2017). In addition, the circular form of LINC-PINT could translate a functional peptide to suppresses the proliferation of glioblastoma cells (Zhang et al., 2018). Although it is reported by a recent study that LINC-PINT is downregulated in melanoma tissues and inhibited cell proliferation through downregulating lncRNA BANCR (Huang et al., 2019), whether LINC-PINT has novel functions with diverse mechanism in human melanoma still remains to be identified.

In this study, it’s our aim to identify the potential regulation role of LINC-PINT in melanoma progression. Through gain- and loss-function experiments *in vitro* and *in vivo*, we found that overexpression of LINC-PINT inhibited the progression of human melanoma. Mechanistically, we showed that LINC-PINT recruited the enhancer of zeste homolog 2 (EZH2) protein to the promoters of CDK1, CCNA2, AURKA, and PCNA gene, leading to H3K27 trimethylation and epigenetic silencing of target genes. Our findings elucidated the tumor-suppressive role of LINC-PINT in human melanoma and unveiled its molecular mechanism underlying tumor progression which might thereby suggest a novel therapeutic strategy for melanoma.

## Materials and Methods

### RNA Preparation, Reverse Transcription and Quantitative Real-Time PCR

Total RNA was extracted from malignant melanoma cell lines and normal control cells using TRIzol Reagent (Invitrogen, Carlsbad, CA, United States) and then cDNA was synthesized using PrimeScript RT Master Mix (Takara, Dalian, China) following the manufacturer’s protocol. Real-time PCR analyses were performed using Power SYBR Green PCR Master Mix (Applied Biosystems, Irvine, CA, United States) on an ABI 7500 real-time PCR system. The glyceraldehyde-3 phosphate dehydrogenase (GAPDH) gene was selected as a reference control. We performed each experiment in triplicate, and listed the primer sets in Supplementary Table 3.

### Cell Lines and Cell Culture

The human malignant melanoma cell line A375 and Mum2B were cultured in DMEM (GIBCO, Carlsbad, CA, United States) supplemented with 10% certified heat-inactivated fetal bovine serum (FBS; GIBCO), penicillin (100 U/mL), and streptomycin (100 mg/mL) at 37°C in a humidified 5% CO_2_ atmosphere. The human conjunctival melanoma cell line CRMM1 was maintained in Ham’s F-12K (Kaighn’s) medium (GIBCO), and the human melanocyte cell line PIG1 was cultured in Medium 254 (GIBCO) with 10% FBS and antibiotics under conditions described above.

### Plasmid Construction, Lentivirus Packaging and Transfection

The pCDNA3.1 vector (Genechem Technology Co., Shanghai, China) was used in our system. To overexpress LINC-PINT, the LINC-PINT sequence was generated by PCR and cloned into the pcDNA3.1 vector. For lentivirus packaging, the Lipofectamine 2000 reagent (Invitrogen, Carlsbad, CA, United States) was incubated with Opti-MEM I Reduced Serum Medium (GIBCO) and used to transfect 293T cells with 3 μg pCDNA3.1-LINC-PINT or pCDNA3.1-vector plasmids or 3 μg pMD2. D plasmids or 6 μg PsPax plasmids. 48 and 72 h after transfection, the supernatant containing the virus was collected, filtered and concentrated. 24 h prior to the lentiviral transfection, the cells were seeded at 3.0 × 10^5^ cells per well in a 60 mm plate. The next day, an optimal volume of lentivirus was added into the culture medium and supplemented with 8 ng/ml polybrene (Sigma-Aldrich, St. Louis, MO, United States), and the cells were maintained in the virus-containing medium for 48 h. Stable cell lines were selected by incubating with 4 μg/ml puromycin (InvivoGen, San Diego, CA, United States) for 2 weeks.

### Western Blot

In brief, cells were harvested, rinsed and lysed with lysis buffer, and the total protein concentration was measured using a BCA protein assay kit (Beyotime Institute of Biotechnology, China). Protein samples were separated using sodium dodecyl sulfate-polyacrylamide gel electrophoresis (SDS-PAGE) in 7.5% (*w*/*v*) polyacrylamide gels and then transferred to polyvinylidene fluoride membranes (PVDF membranes; Millipore, Bedford, MA, United States). After the membranes were blocked with 5% BSA for 1 h at room temperature, they were incubated with the primary antibodies anti-EZH2 (Abcam, Cambridge, MA, United States), anti-CDK1 (Abcam), anti-CCNA2 (CST, Danvers, MA, United States), anti-AURKA (Abcam), anti-PCNA (CST), or anti-β-Actin (Sigma-Aldrich) at 4°C overnight. The horseradish peroxidase-conjugated secondary antibodies (mouse IgG and rabbit IgG, CST) were utilized for 1-h incubation at room temperature. β-actin served as a reference control. The band signals were visualized and quantified using the ECL-PLUS/Kit (Millipore).

### CCK8 Assay

To evaluate cell proliferation capability, the CCK8 (Cell Counting Kit-8) colorimetric assays was utilized. Cells were seeded at 2.0 × 10^3^ cells per well into the flat-bottomed 96-well plates with 100 μl culture medium. Then, 10 μl of CCK8 solution (Dojidon, Kumamoto, Japan) was added to the wells, and the samples were incubated at 37°C in 5% humidified CO_2_ atmosphere. A microplate reader (ELX800, BioTec, Winooski, VT, United States) was employed to measure the absorbance of samples at 450 nm for four consecutive days, as previously described. We performed each independent experiment three times and presented the results as the mean ± SD.

### Soft Agar Formation Assay

Soft agar formation assay was conducted as described in our previous study. LINC-PINT-oe or Mock cells were harvested, counted and resuspended in 1.0 ml 0.3% agar complete medium and 1.0 × 10^3^ cells were seeded into six-well plates embedded with 1.0 ml 0.6% agar complete medium layer. After 3–4 weeks of incubation, the colonies were stained with 1% crystal violet, sufficiently washed with PBS, then they were counted and photographed. To calculate the colony formation rate, the number of colonies generated in Mock cells group were set to one.

### Cell Cycle Analysis

Cells were harvested, washed once with cold PBS and fixed with pre-cold 75% ethanol at −20°C overnight. After incubating with RNase A (Qiagen, Hilden, German) in 37°C for 30 min, the cells were stained with 50 μl/ml PI and treated with 0.5% Triton PBS. Then, the cells were incubated with an anti-H3 antibody (Abcam) at a 1:100 dilution. The stained cells were subjected to analysis by flow cytometry facility (Guava easyCyte HT from Millipore).

### Rapid Amplification of cDNA Ends Assay (RACE)

Total RNA was extracted by TRIzol plus RNA Purification Kit (Invitrogen). RACE PCR products were obtained using Platinum PCR Supermix High Fidelity (Invitrogen) and separated using a 1.5% agarose (Sigma) gel. A gel extraction kit was utilized to extract the gel products, which were then cloned into a pGM-T vector and sequenced. The specific 3′ RACE and 5′ RACE primers are listed in Supplementary Table 3.

### Chromatin Isolation by RNA Purification (ChIRP)

A Magna ChIRP RNA Interactome Kit (Millipore) was used to perform ChIRP experiment according to the manufacturer’s protocols. A group of 3′ end Biotin-TEG modified DNA probes targeting LINC-PINT was synthesized and utilized. A total of 5 × 10^8^ cells were cross-linked with 3% formaldehyde and sonicated for the hybridization reaction. The sequences of the probes are available in Supplementary Table 3.

### RNA-Chromatin Immunoprecipitation (RIP)

The EZ-Magana RIP kit (Millipore) was utilized to perform RIP experiment per the manufacturer’s previously reported protocol. A total of 1.0 × 10^7^ cells were lysed with RIP lysis buffer and subjected for co-immunoprecipitation with anti-EZH2 (Active Motif, Carlsbad, CA, United States) or normal mouse IgG antibody (Millipore). Then the retrieved RNA was analyzed by reverse transcription PCR.

### Chromatin Immunoprecipitation (ChIP)

For ChIP assay, the EZ-Magna ChIP A/G kit (Millipore) was used following the instructions as previously described by the manufacturer. The anti-EZH2 (Active Motif) and anti-H3K27me3 (Active Motif) were applied. Anti-normal mouse IgG (Millipore) and anti-RNA polymerase-II (Abcam) were used as negative control, or positive control, respectively. Primers for ChIP-qPCR are listed in Supplementary Table 3.

### DNA Microarray Analysis and Bioinformatics Analysis

For a gene expression study, we purified and hybridized three independent replicates of RNA samples from A375 cells with or without LINC-PINT overexpression to microarray gene chips. In this experiment, the GeneChip PrimeView Human Gene Expression Array (Affymetrix, Santa Clara, CA, United States) was employed. In brief, total RNA samples from A375 Mock cells and A375 LINC-PINT oe cells was extracted using TRIzol reagent (Invitrogen, United States) and quantified by NanoDrop 2000 (Thermo Fisher Scientific, Waltham, MA, United States). 0.5 μg purified RNA was transcribed to cDNA. The Agilent RNA 6000 Nano Kit (Agilent Technologies, Santa Clara, CA, United States) was used to assess the RNA integrity by the Agilent 2100 Bioanalyzer. The GeneChip 3’ IVT labeling kit (Affymetrix) was used to synthesize biotin-labeled RNA, which were then hybridized onto the microarrays. After the sample labeling, microarray hybridization and washing steps were conducted following the manufacturer’s instructions, the arrays were directly scanned by the Affymetrix Scanner 3000 (Affymetrix). The subsequent microarray data processing was done as previously described (Lu et al., 2017). Differentially expressed genes were identified by a threshold of three-fold change (*p*-value < 0.05).

### Tumor Xenograft Model in Nude Mice

Four-week-old male BALB/c nude mice were maintained in specific pathogen-free (SPF) animal room and used in tumor xenograft assays. A total of 1.0 × 10^7^ A375 cells transfected with or without LINC-PINT lentivirus were subcutaneously injected into the right armpit of the BALB/c nude mice (*n* = 5 for each group). The tumor volume [length (mm) × width (mm)^2^/2] of each mouse was measured every five days for twenty-five consecutive days. Afterward, the mice were euthanized and the tumors were harvested, evaluated and photographed. For the *in vivo* metastasis assay, the nude mice were deeply anesthetized and total of 2.0 × 10^6^ A375 or Mum2B cells transfected with *Luc*-tag from LINC-PINT overexpression or Mock group were injected through the caudal vein of each mouse (*n* = 3 for each group). We utilized live animal BLI system to monitor tumor growth and lung metastases. All the mice were sacrificed after 3 weeks and the lungs were carefully resected, fixed and examined for metastases via haematoxylin and eosine (HE) staining.

The animal experiments were carried out in strict accordance with the guidelines of the Shanghai Jiao Tong University School of Medicine Animal Care and Use Committee, by whom the protocols were also approved (permit number: HKDL [2014]70, 25 February 2014).

### Statistical Analyses

For all of the results, the data are presented as the mean ± SD, and a *p*-value less than 0.05 was considered statistically significant. The differences between two groups were compared by unpaired two-sided Student’s *t*-test or ANOVA. We performed the analyses with IBM SPSS Statistics 20 (Chicago, IL, United States).

## Results

### LINC-PINT Was Lowly Expressed in Melanoma Tumor Tissues and Cell Lines

**FIGURE 1:**
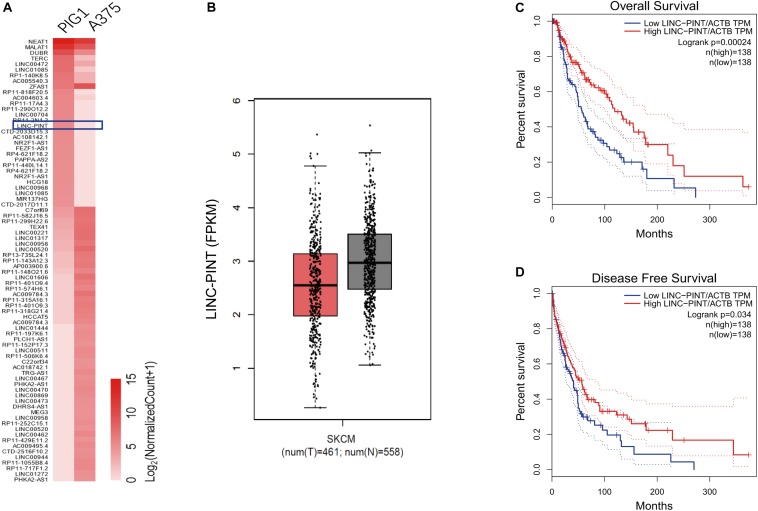
LINC-PINT was lowly expressed in melanoma tumor tissues and cell lines. **(A)** Hierarchical cluster plot showed the top 50 up- and 30 down-regulated lncRNAs (fold change >2, *p* < 0.05). The blue square denotes LINC-PINT **(B)** The expression of LINC-PINT in melanoma tissues (*n* = 461) versus adjacent normal tissues (*n* = 558) from GEPIA database (Gene Expression Profiling Interactive Analysis database; gepia.cancer-pku.cn). The whiskers indicate means ± SD in the plots. **(C–D)** Kaplan–Meier survival analysis of patient overall survival **(C)** and disease-free survival **(D)** according to LINC-PINT levels in melanoma tissues.

We used high-throughput RNA-sequence analysis to identify the lncRNAs that were differentially expressed between melanoma A375 cells and normal control PIG1 cells. We found that LINC-PINT was one of the most downregulated lncRNAs in melanoma cells (Figure 1A). Moreover, LINC-PINT expression level was also significantly low in melanoma tissues compared with adjacent normal tissues (Figure 1B). Notably, survival analysis showed that low LINC-PINT expression was prominently correlated with poor overall survival (Figure 1C) and disease-free survival (Figure 1D) for melanoma patients. These data indicated that LINC-PINT might play a key regulatory role in melanoma progression.

### Identification and Cellular Distribution of LINC-PINT in Melanoma Cells

**FIGURE 2:**
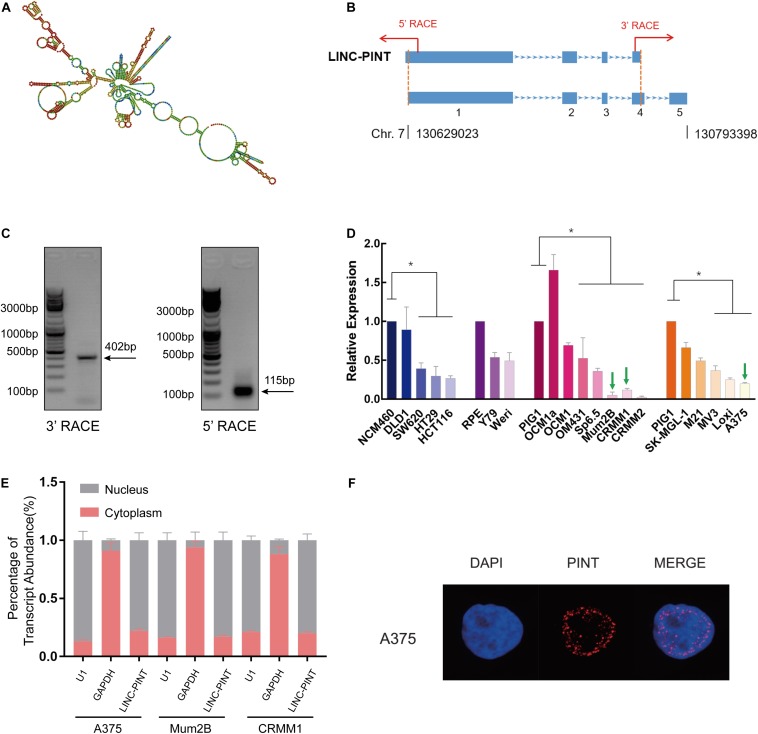
Identification and cellular distribution of LINC-PINT in melanoma cells. **(A)** Secondary structure of LINC-PINT predicted by RNAfold web server (http://rna.tbi.univie.ac.at/cgi-bin/RNAWebSuite/RNAfold.cgi). **(B)** Schematic illustration of the primers for RACE assay. **(C)** Agarose gel electrophoresis of PCR products generated by 3’- (left) and 5’- (right) RACE technologies. **(D)** Real-time PCR analysis of LINC-PINT expression in different cell lines. LINC-PINT presented lower expression in a series of tumor cells than in normal gastrointestinal cells (NCM460), retinal pigment epithelium cells (RPE) and normal skin cells (PIG1) ^∗^*p* < 0.05. **(E)** Cell nuclear/cytoplasmic fraction analysis and real-time PCR confirmed LINC-PINT was expressed mainly in the nucleus; U1 and GAPDH RNA served as positive controls for the nuclear and cytoplasmic fractions, respectively. **(F)** RNA FISH analysis shows that LINC-PINT was located predominantly in the nucleus of A375 cells.

Then we aimed to identify the biological characteristics of LINC-PINT in melanoma. We predicted the secondary structure of LINC-PINT in the RNAfold web server (Figure 2A). Furthermore, total RNAs extracted from melanoma cells (A375) was used to clone the full-length of LINC-PINT transcripts by 5’- and 3’- RACE technologies (Figure 2B). As shown in Figure 2C, both 3’-RACE and 5’-RACE results showed that only one band was presented, indicating that there are only one LINC-PINT isoform exists in melanoma cells (Figure 2C). According to the National Center for Biotechnology Information (NCBI) database, the transcript of LINC-PINT previously reported was 1173-bp in length with four exons. In our study, however, we identified a novel 1430-bp transcript with five exons through the rapid amplification of cDNA ends (RACE) detection. More precisely, exon one of the novel transcript had an additional 12-bp fragment at the 5-terminus, and exon four was extended by 279-bp. Compared with the predict sequence, this novel transcript also had an additional poly-A tail at the 3-terminus (Supplementary Figure 1). We then examined LINC-PINT expression in different tumor cells. The expression levels of LINC-PINT in melanoma cells were significantly low (Figure 2D). Thus, we selected melanoma cell lines A375, Mum2B and CRMM1 to test whether LINC-PINT overexpression could alter the tumor behavior. The biological function of lncRNAs is strongly associated with their subcellular localizations. Thus, cellular fractionation assay was conducted and determined that LINC-PINT distributed mainly in the nucleus of melanoma cells (Figure 2E). RNA fluorescence *in situ* hybridization (RNA-FISH) further confirmed that LINC-PINT was enriched in the nuclear fraction (Figure 2F).

### LINC-PINT Inhibited Melanoma Progression *in vitro*

**FIGURE 3:**
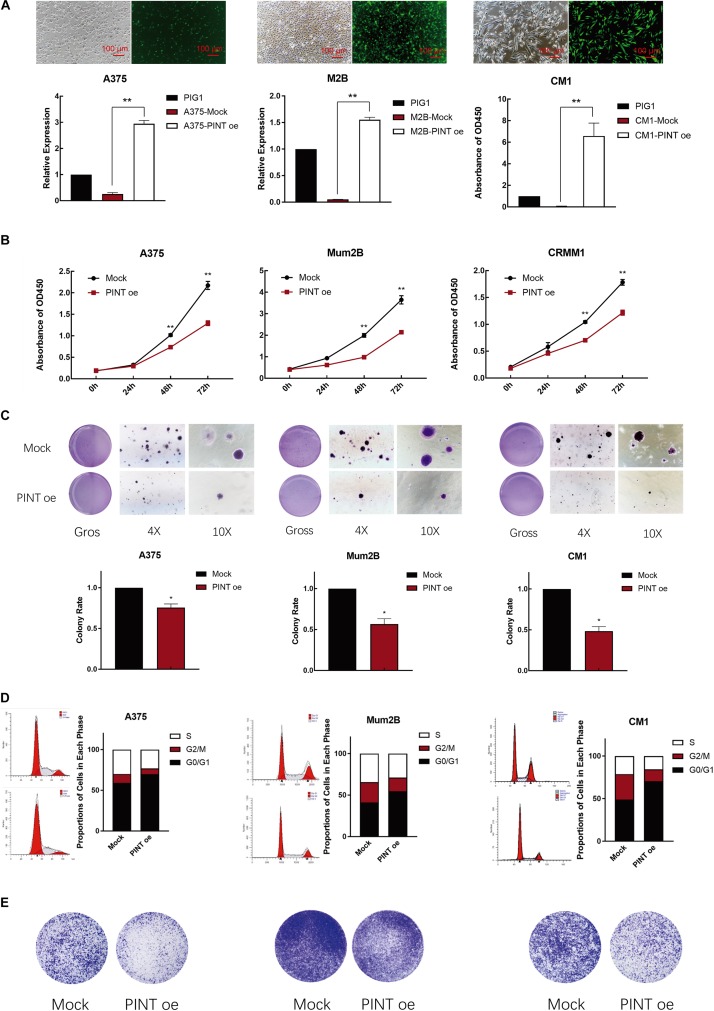
LINC-PINT inhibited melanoma progression *in vitro.* **(A)** The LINC-PINT overexpression plasmid was stably transfected into A375, Mum2B and CRMM1 cells, and the plasmid also contained the EGFP tag. Scale bars: 100 μm. Real-time PCR results showed significantly higher expression of LINC-PINT in A375-PINT oe, M2B-PINT oe and CM1-PINT oe cells. ^∗∗^*P* < 0.01. **(B)** CCK8 assay was performed to assess cell proliferation in the Mock and LINC-PINT overexpression melanoma cells. ^∗∗^*p* < 0.01. **(C)** Colony count statistics demonstrated tumor formation ability. The colony count statistics showed a significant reduction in the numbers of LINC-PINT overexpressed A375, Mum2B and CRMM1 cells. The colony numbers were determined from three independent soft agar plates. ^∗∗^*P* < 0.01. **(D)** Cell cycle analysis by flow cytometry was performed to determine the percentage of cells in different cell cycle phases. The percentage of cells in G0/G1 phase increased after LINC-PINT overexpression in A375, Mum2B and CRMM1 cells. All histograms showed the percentage (%) of cell populations from one independent experimental group. **(E)** The migration and invasion abilities of LINC-PINT overexpressed melanoma cells were detected by transwell assay.

To investigate whether the tumor behavior could be significantly altered by this novel transcript of LINC-PINT, we first overexpressed LINC-PINT by using control cell lines, which was transfected with virus carrying an empty pcDNA3.1 vector. Using EGFP as a tracking marker, we then observed green fluorescence in A375, Mum2B and CRMM1 cells. We detected that LINC-PINT was successfully overexpressed in these melanoma cells by real-time PCR (Figure 3A). CCK8 assay showed that cell proliferation was significantly suppressed in LINC-PINT-overexpressed melanoma cells (Figure 3B). Consistently, the colony formation of melanoma cells was decreased after overexpressing LINC-PINT. We also observed that the LINC-PINT-overexpressed melanoma cells formed smaller colonies (Figure 3C). Moreover, we performed flow cytometry assay to determine whether LINC-PINT was involved in cell cycle regulation and found that LINC-PINT overexpression induced G0/G1 cell cycle arrest in melanoma cells (Figure 3D). Furthermore, transwell assay showed that LINC-PINT overexpression also inhibited the migration ability of melanoma cells (Figure 3E).

### LINC-PINT Inhibited Melanoma Progression *in vivo*

**FIGURE 4:**
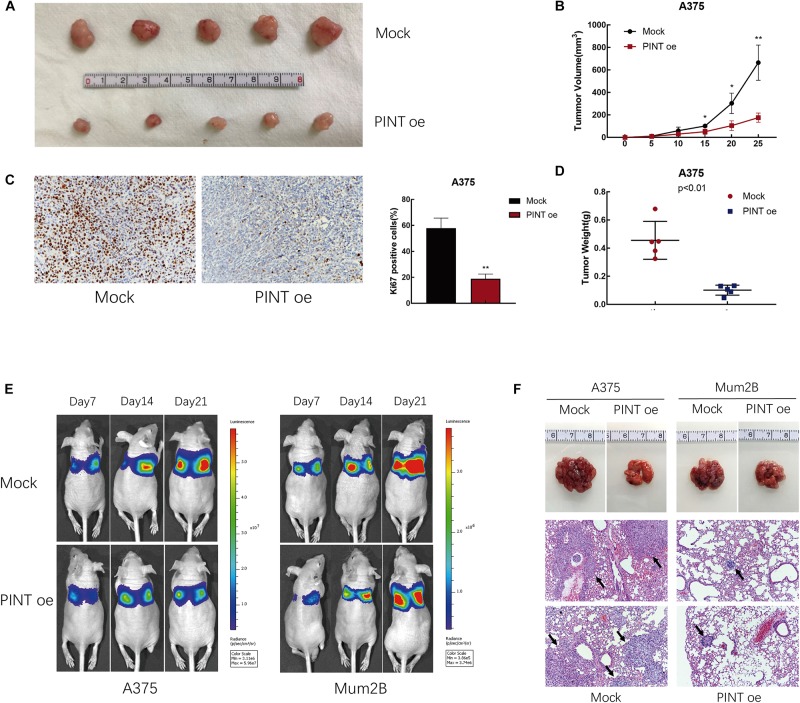
LINC-PINT inhibited melanoma progression *in vivo.* **(A)** Mock and PINT oe A375 cells were injected into the nude mice. Tumors derived from cells with (lower panel) or without (upper panel) LINC-PINT overexpression were removed from the mice. **(B)** Tumor volume was evaluated every 5 days after the injection of Mock (*n* = 5) and PINT oe (*n* = 5) A375 cells for 25 consecutive days. ^∗^*p* < 0.05, ^∗∗^*p* < 0.01. **(C)** Ki-67 staining of Mock and PINT oe tumor tissues. **(D)** The weights of Mock (*n* = 5) and PINT oe (*n* = 5) A375 tumors. **(E)** Effect of LINC-PINT on tumor metastasis in a lung metastasis mouse model. Mock and PINT oe A375 and Mum2B cells were injected into the caudal vein of nude mice (*n* = 3 for each group). **(F)** Representative lung tissues and their HE-stained sections (100× magnification) are shown. The black arrows showed the metastases.

To investigate the ability of LINC-PINT to suppress tumor formation *in vivo*, we established a xenograft model in nude mice using A375 cells. We injected A375 and LINC-PINT-overexpressed A375 cells into nude mice. Then, we evaluated the size of the resultant tumors every 5 days for 25 days. We found that LINC-PINT overexpression notably repressed tumor progression. The tumor volumes in the overexpression group were significantly reduced compared with those of the controls (Figures 4A,B). Immunohistochemistry staining showed that compared with those from control group, tumors derived from LINC-PINT-overexpressed group exhibited lower expression of proliferation marker Ki67 (Figure 4C). Moreover, the tumor weights from LINC-PINT-overexpressed group were also significantly reduced (Figure 4D). In addition, we assessed the impact of LINC-PINT on metastasis ability *in vivo* using a lung metastasis mouse model. The results revealed that LINC-PINT overexpression noticeably inhibited melanoma metastasis (Figures 4E,F). Taken together, these data were consistent with *in vitro* results.

### Identifying the Target Genes of LINC-PINT in Melanoma Cells

**FIGURE 5:**
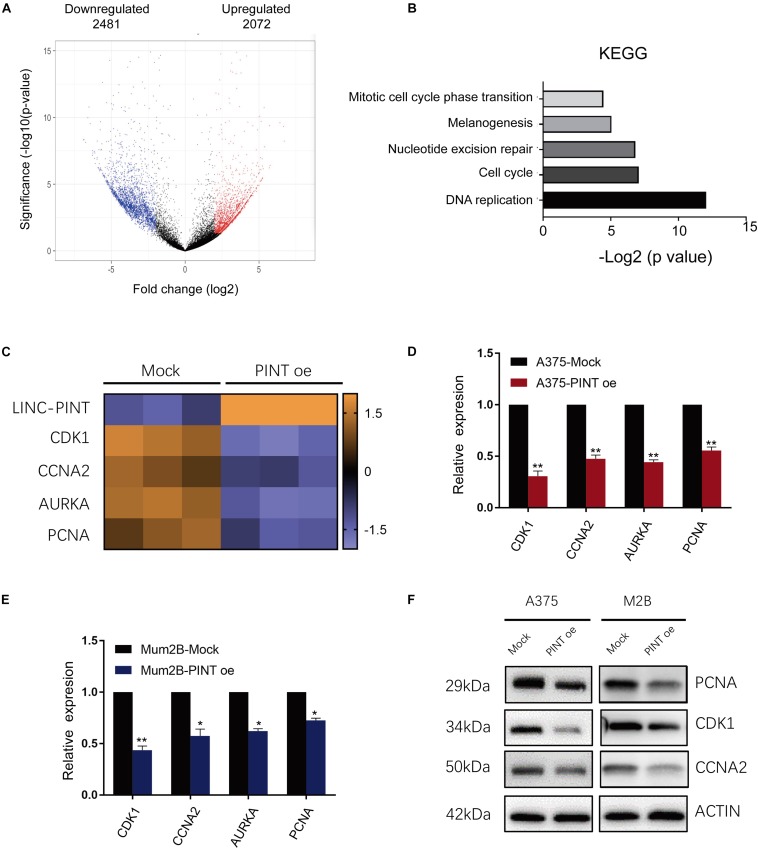
The target genes of LINC-PINT were identified in melanoma cells. **(A)** Volcano plots of differentially expressed gene. The *X* axis represented log fold changes. The *Y* axis represented log *p*-values. The red points denoted the significantly upregulated genes and blue points denoted the significantly downregulated genes. **(B)** KEGG analysis of differentially expressed genes between Mock and PINT oe A375 cells. **(C)** Heat map showed the differentially expressed genes related to cell cycle progression. **(D,E)** The downregulated cell cycle genes in the microarray were verified in A375 and Mum2B cells by real-time PCR. ^∗^*p* < 0.05, ^∗∗^*p* < 0.01. **(F)** The expression of LINC-PINT target genes was detected by western blot. Actin were used as internal controls.

To comprehensively analyze the tumor-suppressive regulatory effect of LINC-PINT on gene expression, we performed a microarray analysis to profile gene expression in melanoma cells with or without LINC-PINT overexpression. The results showed that 2481 transcripts were downregulated while 2072 transcripts were upregulated in LINC-PINT-overexpressed A375 cells (Figure 5A). We then performed Kyoto Encyclopedia of Genes and Genomes (KEGG) analysis and found that DNA replication and cell cycle pathways were the highest affected biological processes after LINC-PINT overexpression in A375 cells (Figure 5B). Using hierarchical cluster analysis, we found that the expression of several genes (CDK1, CCNA2, AURKA, PCNA) involving in the cell cycle and tumorigenesis were reduced significantly (Figure 5C). We then validated the expression of these genes by real-time PCR (Figures 5D,E) and western blot (Figure 5F). Collectively, these data suggested that LINC-PINT regulated melanoma progression by modulating the expression of a series of cell cycle genes.

### LINC-PINT Recruited EZH2 to Inhibit Gene Expression

**FIGURE 6:**
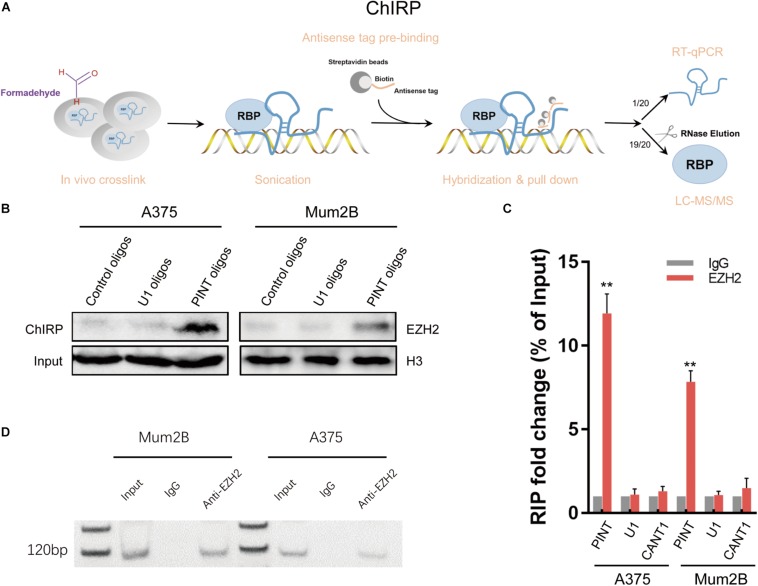
LINC-PINT recruited EZH2 to inhibit gene expression. **(A)** Introduction of the ChIRP method. **(B)** EZH2 was detected from the retrieved ChIRP protein of A375 and Mum2B cells by western blot. PINT oligos indicated the biotinylated antisense oligonucleotides against LINC-PINT. Control oligos indicated the scrambled oligonucleotides, and U1 oligos were selected as a negative control. **(C)** The interaction of LINC-PINT with EZH2 was verified by RIP assay. The values are normalized to input. ^∗∗^*p* < 0.01. LncRNA CANT1 and U1 RNA served as negative controls. **(D)** Agarose gel electrophoresis of RIP products.

**FIGURE 7:**
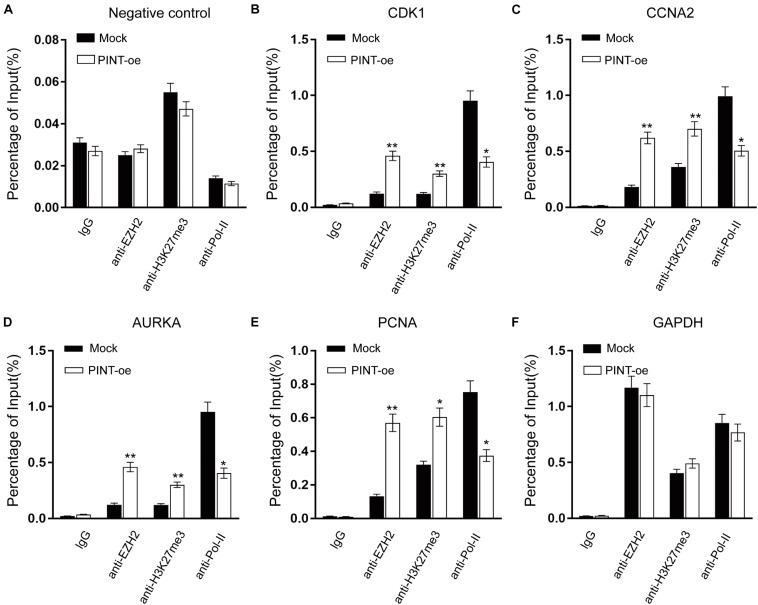
Interactions between epigenetic modifiers and the promoter regions of LINC-PINT target genes. **(A–F)** ChIP analysis of IgG, EZH2, H3K27me3, and RNA polymerase-II showed the status of candidate LINC-PINT target genes in A375 cells with or without LINC-PINT overexpression. The values were normalized to input. ^∗^*p* < 0.05; ^∗∗^*p* < 0.01.

LncRNAs are reported to fulfill their functions through active interactions with RNA binding proteins (Guttman and Rinn, 2012; Geisler and Coller, 2013). To explore the molecular mechanism by which LINC-PINT affects gene expression, we sought to identify proteins that were interacting with LINC-PINT by chromatin isolation by RNA purification (ChIRP) experiment (Figure 6A; Supplementary Figure 2). LINC-PINT-binding proteins was then identified by mass spectrometry (Supplementary Tables 1, 2). EZH2 was the only functional protein distributed in the nucleus that was binding to LINC-PINT both in A375 and Mum2B. Tri-methylation of lysine 27 on histone 3 (H3K27me3) by the methyltransferase EZH2, as a part of PRC2, is one of the most important epigenetic mechanism of gene silencing (Hirukawa et al., 2018). The ChIRP-MS results showed that EZH2 protein was enriched by LINC-PINT probes, but not the negative control probes, as further confirmed by western blot (Figure 6B). The interaction of LINC-PINT with EZH2 was further validated by RNA immunoprecipitation (RIP) experiment (Figures 6C,D). Then we performed a chromatin immunoprecipitation (ChIP) assay in Mock and LINC-PINT overexpressed A375 cells to confirm the interactions between epigenetic modifiers and the promoter regions of LINC-PINT target genes. As expected, LINC-PINT overexpression generated decreased location of EZH2, H3K27me3, and RNA polymerase-II levels in the promoter regions of CDK1, CCNA2, AURKA, and PCNA, but not in the negative control and GAPDH, indicating that LINC-PINT overexpression might directly result in downregulation of these genes (Figures 7A–F).

## Discussion

Although mutation of some genes, such as BRAF, NRAS, and TP53, have been identified as risk factors for melanoma progression (Byron et al., 2012; Posch et al., 2013; Lissanu Deribe et al., 2016; Hayward et al., 2017; Ojha et al., 2019), our knowledge of molecular mechanism underlying the malignant melanoma remains obscure. Recently, numerous lncRNAs have been discovered in diverse types of tumors through high-throughput RNA sequencing technologies. Growing evidence suggested that lncRNAs may act as epigenetic modifiers to regulate gene expression in tumor initiation and development (Gupta et al., 2010; Yap et al., 2010; Tseng et al., 2014). In melanoma, lncRNA SAMMSON increases its mitochondrial targeting and pro-oncogenic function by interacting with p32, a master regulator of mitochondrial homeostasis and metabolism (Leucci et al., 2016), lncRNA SLNCR1 mediates melanoma invasion through a highly conserved sequence binding to brain-specific homeobox protein 3a (Brn3a) and the androgen receptor (AR). SLNCR1, AR, and Brn3a are specifically required for transcriptional activation of matrix metalloproteinase 9 (MMP9) and increased melanoma invasion (Schmidt et al., 2016). However, our knowledge of lncRNAs in tumors, especially in melanoma, remains limited. Here, we revealed a novel transcript of LINC-PINT as a tumor suppressor to inhibit melanoma progression via recruiting EZH2 to the promoter of cell cycle related genes.

Polycomb repressive complex 2 (PRC2) is recently reported to play a central role in controlling critical cellular processes including maintaining stem cell pluripotency, promoting cell proliferation and mediating myogenic differentiation (Boyer et al., 2006; Xu et al., 2018). Gene silencing mediated by polycomb is considered to depend mostly on regulation of chromatin structure, in part through post-translational modification (PTM) of histones (Margueron and Reinberg, 2011). In the past few years, enhancer of zeste homolog 2 (EZH2), the catalytic subunit of Polycomb repressive complex 2 (PRC2), has aroused broad research interest because of its multiple roles in the development of many types of cancer (Margueron and Reinberg, 2011; Di Croce and Helin, 2013; Comet et al., 2016; Kim and Roberts, 2016). EZH2 both fulfills its oncogenic and tumor suppressive roles in a variety of cancers. It is demonstrated that EZH2 is mainly upregulated in solid tumors, melanoma included, which indicates a more aggressive tumor growing pattern and poorer prognosis in most cases. In late stage prostate tumors, EZH2 upregulation is related to gene amplification, whereas its expression is profoundly affected by the BRAF (V600E) mutation in melanoma (Volkel et al., 2015). *In vivo* study shows that EZH2 overexpression results in intraductal epithelial hyperplasia through inducing β-catenin nuclear accumulation and activating Wnt/β-catenin signaling in mammary epithelial cells (Li et al., 2009). In addition, expression of Ezh2^Y641F^, the most common somatic EZH2 mutation (Y646F in human, Y641F in the mouse), in mouse melanocytes causes melanoma through a vast reorganization of chromatin structure (Souroullas et al., 2016). Meanwhile, potent molecules selectively targeting EZH2 enzymatic activity have been discovered, including EPZ005687, EPZ-6438, EI1, UNC1999, and GSK126, which also exerts significant anti-tumor effects in distinct melanoma subsets (Volkel et al., 2015; Perotti et al., 2019). Epigenetically, PRC2-mediated gene silencing is mainly dependent on the regulation of EZH2-mediated H3K27me3 (Schuettengruber and Cavalli, 2009; Simon and Kingston, 2009). Recently, a growing body of evidence shows that epigenetic factors, like lncRNAs are involved in the EZH2-mediating tumor regulation. LncRNA exerts its biological function mainly by binding to RNA binding protein (RBP) (Gou et al., 2018). The lncRNAs ANCR, HOTAIR, H19, are involved in the recruitment of EZH2 to the specific regulatory regions of its target genes (Davidovich and Cech, 2015). For instance, the long intergenic non-coding RNA HOTAIR generates the trimethylation of H3K27 and epigenetic silencing of metastasis-suppressor genes via recruiting EZH2 to specific target genes (Gupta et al., 2010). LncRNA ANCR interacts with EZH2 to promote its phosphorylation that facilitates EZH2 degradation and suppresses breast cancer progression (Li et al., 2017). In this study, we demonstrated that LINC-PINT was an EZH2-binding lncRNA and play an tumor-suppressive role in melanoma progression. We performed ChIRP assay and mass spectrometry to identify specific association of LINC-PINT with EZH2, which was further confirmed by RIP and Western blot. With more importance, it is confirmed by ChIP assay that LINC-PINT regulated activation of downstream genes by interacting with EZH2, which in turn mediated H3K27me3 at the promoter regions of target genes. Thus, our results demonstrate that LINC-PINT interacts directly with the promoter region of target genes and mediates H3K27me3 to activate transcription via binding to EZH2.

Although the function and structure of LINC-PINT have been studied for more than 4 years, a more comprehensive role of LINC-PINT in regulating the melanoma tumorigenesis still remains to be elucidated. Additionally, recent studies have shown that LINC-PINT plays a vital role in several types of human cancers, melanoma included (Huang et al., 2019). In this manuscript, it is observed that overexpression of LINC-PINT significantly inhibited melanoma proliferation both *in vitro* and *in vivo*. Through further explorations, we identified genes that were mainly suppressed by LINC-PINT, including CDK1, CCNA2, AURKA, and PCNA. These genes have been confirmed as critical regulators in melanoma and might represent therapeutic targets for clinical application. For example, CDK1 was reported to interact with Sox2 and promote tumor initiation in human melanoma (Ravindran Menon et al., 2018), CCNA2 and AURKA inhibitors are now available and has shown encouraging effect for treatment of melanoma (Caputo et al., 2014; George et al., 2019). Taken together, these results suggest LINC-PINT as a multi-potent therapeutic target with great potential.

**FIGURE 8:**
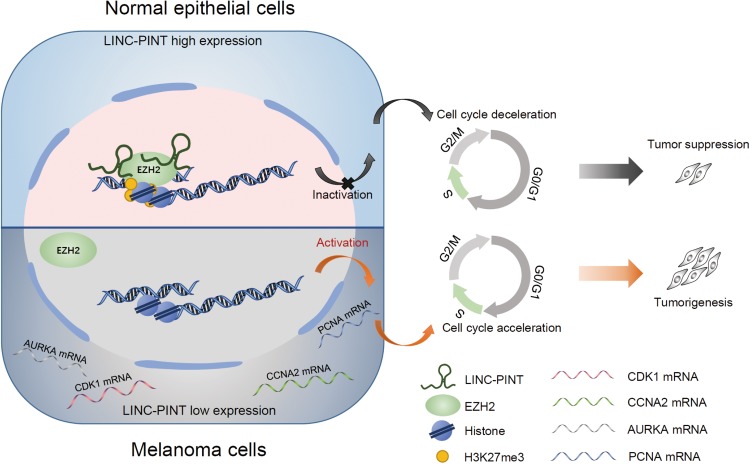
A schematic model of LINC-PINT in epigenetic silencing of target genes in melanoma cells via recruiting EZH2. A novel transcript of LINC-PINT serves as key regulator of melanoma progression. In LINC-PINT highly expressed cells, LINC-PINT inactivated the transcription of CDK1, CCNA2, AURKA, and PCNA genes by the effect of EZH2-mediated H3K27me3, thus leading to the cell cycle deceleration and suppressing the tumorigenesis. However, in LINC-PINT deficient melanoma cells, the epigenetic regulation effect was abolished, subsequently promoting the expression of target genes and contributing to melanoma expansion.

In summary, we identified an lncRNA, LINC-PINT, and proposed a mechanistic model to elucidate its role in the regulation of melanoma progression through EZH2-mediated epigenetic silencing (Figure 8). In the animal xenograft model, LINC-PINT overexpressed melanoma cells represented significant tumor growth inhibition and metastasis reduction effects. Mechanistically, we showed that LINC-PINT recruited EZH2 to the promoter region of its target genes to impede tumor cell proliferation. Therefore, our study elucidates the potential role of LINC-PINT in the development of melanoma and unveils its molecular mechanism underlying tumor progression. Our findings indicate that LINC-PINT could be a potential therapeutic target for human melanoma.

## Conclusion

In summary, LINC-PINT was expressed at remarkably lower levels in melanoma tissues and cell lines. For melanoma patients, lower expression of LINC-PINT was associated with poorer overall survival and disease-free survival. Strikingly, overexpression of LINC-PINT significantly reduced melanoma cells progression via downregulating the potential target genes CDK1, CCNA2, AURKA, and PCNA through recruiting EZH2 protein, which in turn mediated the trimethylation of H3K27 of promoter regions of target genes. Here, we identified the tumor-suppressive role of LINC-PINT in melanoma and uncovered the underlying epigenetic regulatory mechanism.

## Data Availability Statement

The raw data supporting the conclusions of this article will be made available by the authors, without undue reservation, to any qualified researcher.

## Ethics Statement

The animal study was reviewed and approved by the Shanghai Jiao Tong University School of Medicine Animal Care and Use Committee.

## Author Contributions

YX, XH, and FL designed and performed the experiments and drafted the manuscript. LH, JYu, JYa, and SG was responsible for the data analysis. JR, RJ, and XF wrote and approved the manuscript. All of the authors approved the manuscript.

## Conflict of Interest

The authors declare that the research was conducted in the absence of any commercial or financial relationships that could be construed as a potential conflict of interest.
