# Supplementary material for: Long Non-coding RNA LINC-PINT Suppresses Cell Proliferation and Migration of Melanoma via Recruiting EZH2
Source: Front Cell Dev Biol. 2019 Dec 20;7:350. doi: 10.3389/fcell.2019.00350 (PMC6934058; doi:10.3389/fcell.2019.00350)
Supplement: Supplementary file 1 [file Data_Sheet_1.docx]

## **Supplementary files**

**Long Non-coding RNA** **LINC-PINT Suppresses Cell Proliferation and Migration of Melanoma via Recruiting EZH2**

Yangfan Xu^1,2, #^, Huixue Wang^1,2,#^, Fang Li^1,2,#^, LM Heindl ^3^, Xiaoyu He^1,2^, Jie Yu^1,2^, Jie Yang^1,2^, Shengfang Ge ^1,2^, Jing Ruan^1,2*^, Renbing Jia^1,2*^, Xianqun Fan^1,2*^

^1^ Department of Ophthalmology, Ninth People’s Hospital, Shanghai JiaoTong University School of Medicine, Shanghai, P.R. China

^2^ Shanghai Key Laboratory of Orbital Diseases and Ocular Oncology, Shanghai, P.R. China

^3^ Zentrum für Augenheilkunde, Universität zu Köln

^#^ These authors have contributed equally to this work

^*^ Corresponding authors:

Correspondence to XianqunFan, Department of Ophthalmology, Shanghai Ninth People's Hospital, Shanghai JiaoTong University School of Medicine, Shanghai, P.R. China. E-mail: fanxq@sjtu.edu.cn

Renbing Jia: renbingjia@sjtu.edu.cn

Jing Ruan: drjruan@163.com

Telephone: 86-21-23271699

**Figure legend**

Supplemental information includes 2 figures and 3 tables.

**Supplementary Tables**

**Supplementary Table 1.** ChIRP-MS identified LINT-PINT specifically binding proteins in A375

**Supplementary Table 2.** ChIRP-MS identified LINT-PINT specifically binding proteins in Mum2B

**Supplementary Table 3.** Primers, oligos, shRNAs and probes used in the experiment

**Supplementary figure legends**

**Figure S1. 5’ and 3’ RACE of LINC-PINT**

Sequences of 5’ and 3’ RACE. 3’ RACE result indicated that LINC-PINT contain a poly (A) tail of 23 As.

**Figure S2. ChIRP-MS analysis of LINC-PINT-interacting proteins.**

The protein peptides isolated by ChIRP. U1 was selected as control, and scrambled oligos were selected as negative controls.

**Supplementary Figure 1**

**
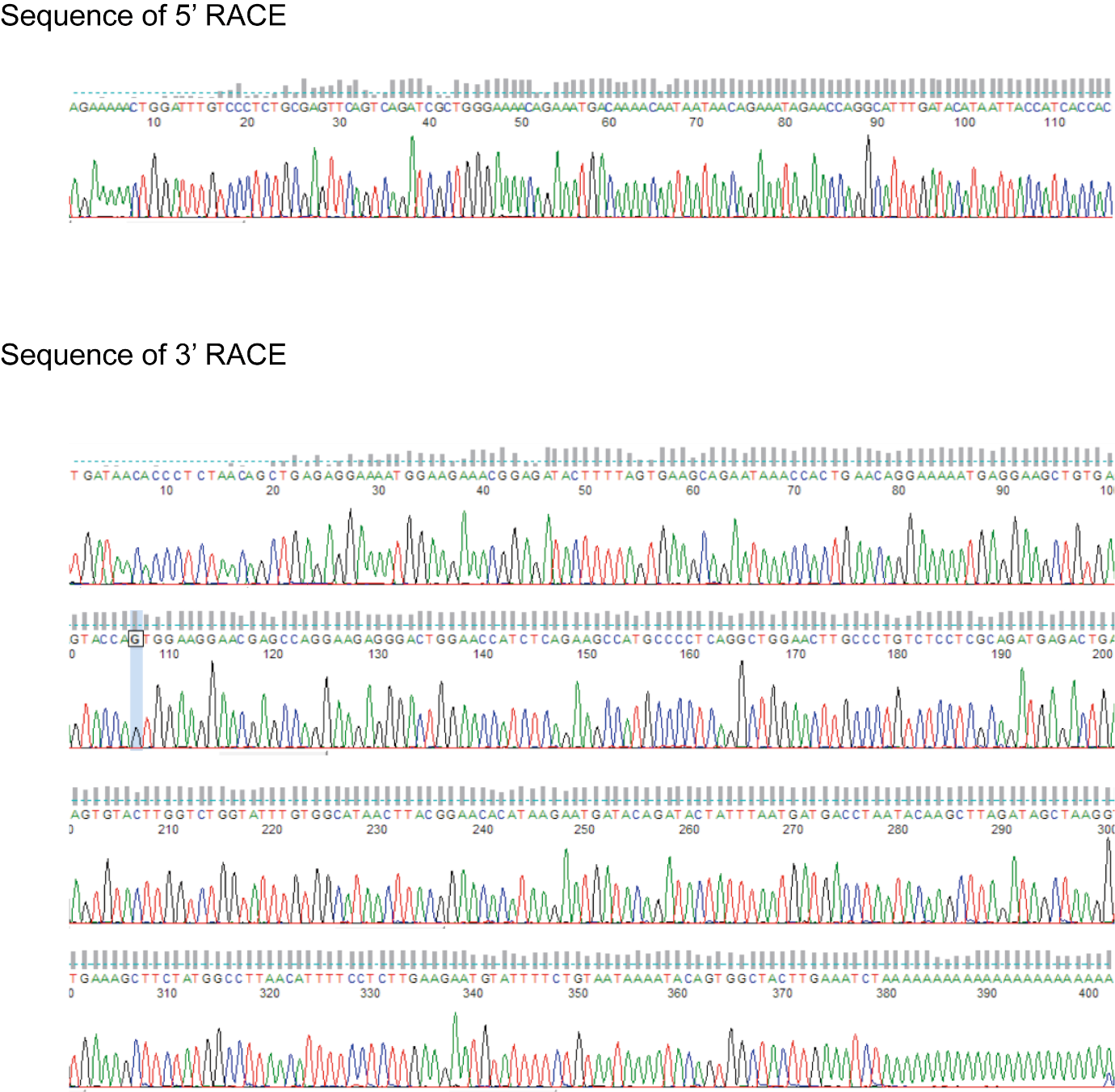
**

**Supplementary Figure 2**


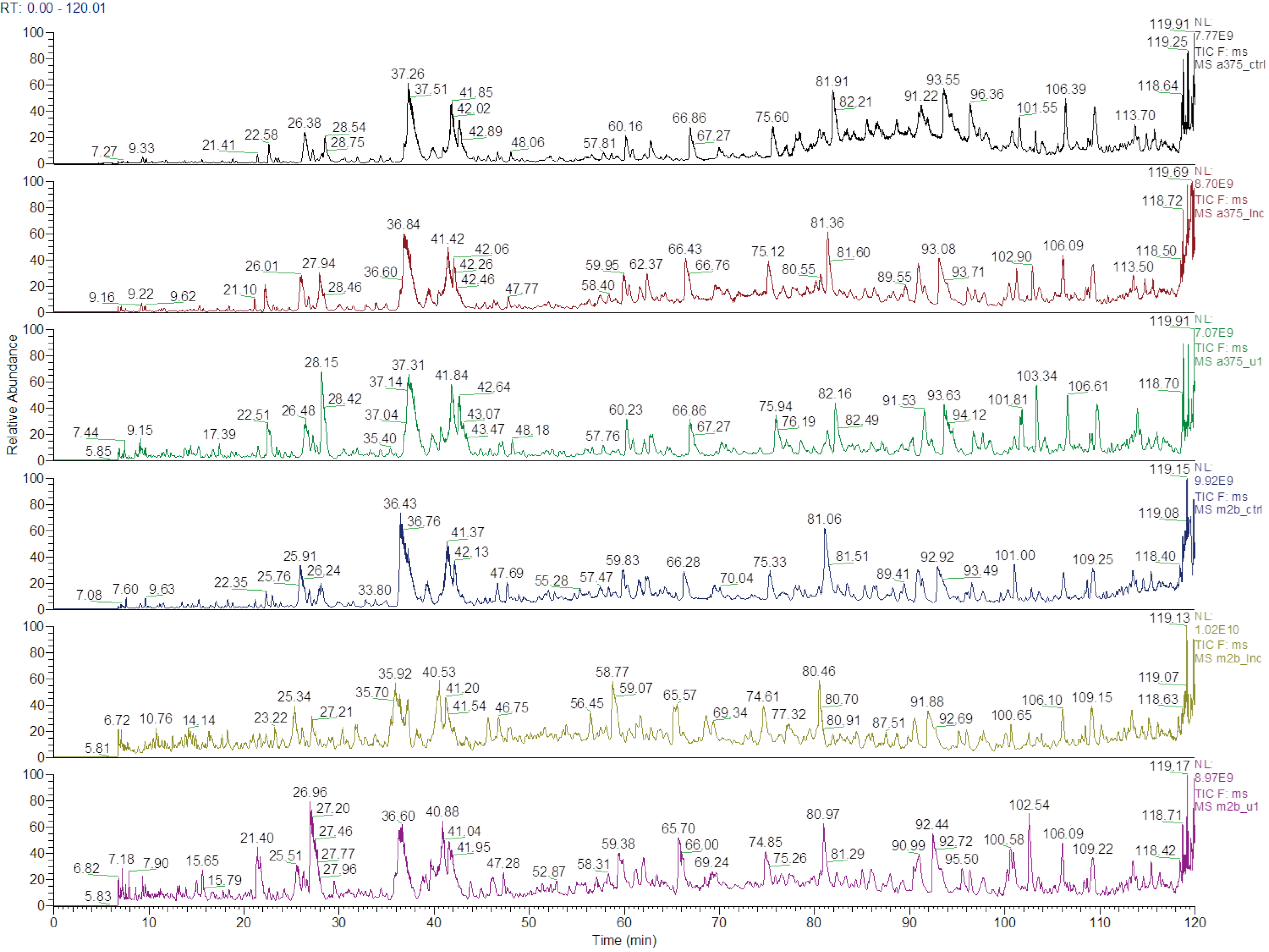


**Supplementary Table 1.** ChIRP-MS identified LINC-PINT specifically binding proteins in A375

| **Gene** | **iBAQ** | **Unique.**  **peptides**  **LINC-PINT** | **Unique.**  **peptides**  **control** | **Rank** | **Score** |
| --- | --- | --- | --- | --- | --- |
| KHSRP  HNRNPM  YBX1  EHZ2  RPL4  RPL10 | 64405000  92550000  77578000  20331000  99963000  40997000 | 35  32  12  21  21  13 | 1  2  1  2  1  1 | 28  9  12  16  21  7 | 240.29  313.67  241.07  323.31  271.38  285.72 |

**Supplementary Table 2.** ChIRP-MS identified LINC-PINT specifically binding proteins in Mum2B

| **Gene** | **iBAQ** | **Unique.**  **peptides**  **LINC-PINT** | **Unique.**  **peptides**  **control** | **Rank** | **Score** |
| --- | --- | --- | --- | --- | --- |
| ACSL3  RPS13  HSPD1  CANX  RPL15  EHZ2  NCL  SSR4  RPS5  ACTN4 | 3778000  48590000  102600000  27634000  54433000  20331000  95178000  19278000  20474000  91409000 | 11  17  48  23  12  14  36  9  7  59 | 0  2  6  3  1  1  4  0  0  5 | 19  33  3  14  11  8  29  35  27  19 | 199.12  251.18  227.01  291.05  272.55  264.69  241.32  216.41  231.16  218.22 |

**Supplementary Table 3.** Primers, oligos, and probes used in the experiment

| **Gene** | **Forward primer** | **Reverse primer** |
| --- | --- | --- |
| LINC-PINT  CDK1  CCNA2  AURKA  PCNA | ACCACTCATTGTTGGCGCATTCACTG  AAACTACAGGTCAAGTGGTAGCC  CGCTGGCGGTACTGAAGTC  GGAATATGCACCACTTGGAACA  CCTGCTGGGATATTAGCTCCA | GTACTCTTCCCACCCCTACTGCCA  TCCTGCATAAGCACATCCTGA  GAGGAACGGTGACATGCTCAT  TAAGACAGGGCATTTGCCAAT  CAGCGGTAGGTGTCGAAGC |

**RT-PCR primers:**

| **Promoter** | **Forward primer** | **Reverse primer** |
| --- | --- | --- |
| CDK1  CCNA2  PCNA  AURKA  GAPDH | ATGGTGTTTAGGTTGGGCT  GCTGGCAGGGAAAATTGAA  CTGGGTCACAGTCAGGTC  TCTGGGGCAGTGAGTTTTCAT  AGAAAGAAAGGGGAGGGGGCA | TGTCTCCTTATGTTGCCCTA  TCCCATTGATTGGGACCCT  CGCCTTGCAGCGGATATC  ACTCCTGCTGGAGCCTAATC  AGCAGGACACTAGGGAGTCAAG |

**CHIP-PCR primers:**

**Primers for LINC-PINT RACE:**

| **Primers for 5' RACE:** | **Primers for 3' RACE:** |
| --- | --- |
| TCCTACTTTGTTTCCCCGGAGAGCA | TGGAGTTTCTCTGCCTTGCTGATCTGA |

**Stellaris probes for RNA-FISH:**

| LINC-PINT | CATTAAATAGTATCTGTATCATTCTTATGTGTTCC |
| --- | --- |

**ChIRP probes for LINC-PINT**

| **Probes** | **Sequence (5’-3’)** | **Probe Locations (start)** |
| --- | --- | --- |
| PINT-P1 | AAATGACATTTCGTGGCTCC | 182 |
| PINT-P2 | CTGTGCTCATGACATAGGAA | 262 |
| PINT-P3 | AGAGGCCTCTCTATGGAAAC | 472 |
| PINT-P4 | TCTCACTCACTTGTTTTTGT | 565 |
| PINT-P5 | CGGCTAAAAGTTGTCCTCCG | 723 |
| PINT-P6 | GCACGTAGATTTGTAGAGCG | 823 |
| PINT-P6 | TTAATAAAATGCCCCAGGGC | 913 |
| PINT-P8 | TAAATTGGCAGGTCTGAGCT | 998 |
| PINT-P9 | GGTTTATTCTGCTTCACTAA | 1080 |
| PINT-P10 | CTTCTGAGATGGTTCCAGTC | 1160 |
| PINT-P11 | GTTATGCCACAAATACCAGA | 1240 |
| PINT-P12 | CCATAGAAGCTTTCACCTTA | 1324 |
